# Relative Contributions of Motor Unit Firing Pattern, Muscle Quantity, and Muscle Quality to Maximum Muscle Strength in Female Patients with Early and Severe Knee Osteoarthritis

**DOI:** 10.1007/s10439-025-03802-2

**Published:** 2025-07-17

**Authors:** Takaya Watabe, Yuichi Nishikawa, Hisashi Naito, Takuya Sengoku, Yuta Taniguchi

**Affiliations:** 1 Section of Rehabilitation, Kanazawa University Hospital, 13-1 Takaramachi, Kanazawa, Ishikawa 920-8641 Japan; 2 Institute of Science & Engineering, Kanazawa University, Kakuma-Machi, Kanazawa, Ishikawa 920-1192 Japan; 3 Faculty of Frontier Engineering, Institute of Science and Engineering, Kanazawa University, Kakuma-Machi, Kanazawa, 920-1192 Japan; 4 Department of Orthopedic Surgery, Graduate School of Medicine Sciences, Kanazawa University, 13-1 Takaramachi, Kanazawa, Ishikawa 920-8641 Japan

**Keywords:** Knee osteoarthritis, Electromyography, Motor unit recruitment, Muscle quantity, Muscle quality

## Abstract

**Purpose:**

While changes in muscle quality and quantity are common concerns as knee osteoarthritis (KOA) progresses, it is equally important to consider the central nervous system’s role in regulating muscle force output. This study aimed to elucidate the firing properties of motor units (MUs), muscle quantity, and muscle quality during submaximal isometric contractions in patients with early and severe KOA.

**Methods:**

This was a prospective study comparing 30 patients with KOA according to the Kellgren/Lawrence grading system divided into early and severe KOA groups. The participants performed ramp-up and sustained contractions at 10%, 30%, and 60% of their maximal voluntary contraction (MVC). High-density surface electromyography signals were recorded in the vastus medialis (VM) and vastus lateralis (VL) muscles on the affected side. Skeletal muscle quantity and quality were assessed using computed tomography based on cross-sectional area (CSA) and computed tomography attenuation value (CTV), respectively.

**Results:**

The CSA of the VM muscle was significantly smaller in the severe KOA group than in the early KOA group (*p* = 0.021). A total of 682 MUs were detected (early KOA, *n* = 348, severe KOA, *n* = 334). The ramp-down phase of the discharge rate (DR) of MUs recruited by the VL muscle in the severe KOA group was significantly lower than that in the early KOA group (*p* = 0.004). In addition, the knee extension torque of patients with early KOA was significantly correlated with the CSA of the VM muscle (*r* = 0.405) and the DR obtained at 30% MVC of the VM (*r* = 0.503) and VL (*r* = 0.610) muscle. Furthermore, the knee extension torque in patients with severe KOA was significantly correlated with the CTV of the VM (*r* = 0.677) and VL (*r* = 0.599) muscles.

**Conclusions:**

In patients with severe KOA, a small CSA of the VM is associated with a characteristic DR pattern depending on the contraction level. The VM and VL muscles fired without a hierarchical pattern in the early and severe KOA groups. These findings suggest that neuromuscular degeneration may occur not only in patients with severe KOA but also in patients with early KOA.

## Background

Knee osteoarthritis (KOA) is a common age-related disease that causes pain, stiffness, and loss of knee function [1]. The increased frequency of KOA worldwide has resulted in increasing medical expenses [2, 3]. KOA is a leading cause of mobility limitations due to pain and stiffness and is often accompanied by quadriceps femoris muscle weakness [4]. This form of osteoarthritis is particularly concerning not only because of its widespread occurrence but also because it affects younger age groups, especially obese women [5]. Reduced strength in the quadriceps femoris muscle is a recognized risk factor for the development of KOA in women, who are 2.6 times more likely to develop this condition than men are [6, 7]. Therefore, maintaining or improving knee extensor strength in KOA patients is important for preventing KOA progression.

Strengthening exercises are a central component of the non-surgical management of KOA, as recommended by Osteoarthritis Research Society International [8]. Traditional KOA exercise therapies heavily emphasize knee extensor strengthening [4]. Anatomical and physiological characteristics of muscles are key determinants of strength [9, 10]. The nervous system has been suggested to be a key determinant of strength/weakness [11]. Skeletal muscle is composed not only of muscle fibers but also of non-contractile tissues, such as adipose and connective which coexist with and support the contractile tissue [12, 13]. Previous studies reported that the quantity of the vastus medialis (VM) and vastus intermedius muscles was lower and the quality of the VM and vastus lateralis (VL) muscles in patients with severe KOA compared to the older adults and patients with early KOA [4, 14]. Because these non-contractile tissues increase with aging and inactivity compared to the older adults and patients with early KOA [15, 16], it is assumed that the accumulation of non-contractile tissues is one factor causing the changes in muscle quality observed in KOA. While changes in muscle quality and quantity are common concerns as KOA progresses, it is equally important to consider the central nervous system’s role in regulating muscle force output. Therefore, it is important to identify the effects of muscle anatomical or neurologic parameters on knee extensor strength in patients with KOA.

Motor unit (MU) firing properties, which differ on the basis of MU recruitment thresholds (RTs), provide insight into the pathway from the central nervous system to muscles [17]. Recent studies utilizing high-density surface electromyography (HD-SEMG) decomposition have explored non-invasive MU identification in various diseases (e.g., parkinson’s disease and amyotrophic lateral sclerosis) [18, 19]. Neuromuscular dysfunction has also been reported in patients with ruptured anterior cruciate ligament reconstruction and presarcopenia via HD-SEMG methods [20, 21]. However, no study has measured the discharge patterns of MUs in KOA patients using HD-SEMG. Therefore, it remains unclear how the discharge patterns of MUs in the VM and VL muscles affect knee extensor strength according to KOA severity.

This study aims to compare the detailed firing properties of individual MUs in the VM and VL muscles during submaximal isometric contractions between early and severe KOA groups. Additionally, the relationships among the MU discharge rate (DR), muscle quality, and quantity were evaluated in the context of the maximal muscle strength. As in previous studies of elderly and presarcopenic individuals [21, 22], we hypothesized that the DR of earlier-recruited MUs would not be greater than that of later-recruited MUs. Additionally, we hypothesized that DR and muscle quality are associated with maximal muscle strength in patients with severe KOA.

## Methods

### Participants

Thirty women with KOA were recruited from the orthopedic surgery department of University Hospital between February 2023 and November 2024. The study protocol and procedures were approved by this University’s Committee on Ethics in Research (Approval Number: 113786) and conformed to the requirements of the Declaration of Helsinki. All patients had apparent radiographic changes indicative of OA in knee and were diagnosed by an orthopedic surgeon as having a Kellgren/Lawrence (K/L) grade ≥ 1. According to the KL grading system, KOA was further divided into early (KL grade 1 and 2) and severe (KL grade 3 and 4) groups. The 2011 Knee Society Score (KSS) was used to assess physical function. The KSS objective knee indicator score ranges from 0 to 100, with lower values indicating more severe symptoms [23]. The inclusion criteria for KOA patients were the ability to live independently; the ability to walk with or without assistive devices; and a pain severity score of ≤ 3 on the Numerical Rating Scale (NRS) at rest and during gait. Patients with a history or diagnosis of the following were excluded: lower limb or back surgery, rheumatoid arthritis, cardiovascular disorders and nervous system problems. The sample size was calculated on the basis of a 95% confidence interval, a power of 0.8, and an effect size of (f) 0.53 (calculated from the separate two-way mixed-model analysis of variance (ANOVA) results of Nuccio et al.) [20]. The analysis resulted in a total sample size of 30.

### Skeletal Muscle Assessment

In this study, we assessed muscle quantity by measuring the cross-sectional area (CSA) and muscle quality by evaluating the computed tomography attenuation value (CTV) on cross-sectional computed tomography images. These measurements were taken at the mid-thigh, specifically at the midpoint between the superior pole of the patella and the inguinal crease, using EV Insite image analysis software (PSP Corporation, Tokyo, Japan). For each patient, manual range selection was applied to the VM and VL muscles, after which the CSA and CTV were calculated for each group. The CSA serves as an indicator of both muscle mass and intermuscular fat, whereas the CTV provides a measure of muscle quality, reflecting the presence of intramuscular fat and intramyocellular lipids. The average CTV for muscle typically ranges between 40 and 100 Hounsfield units [24].

### HD-SEMG Recording

**Fig. 1:**
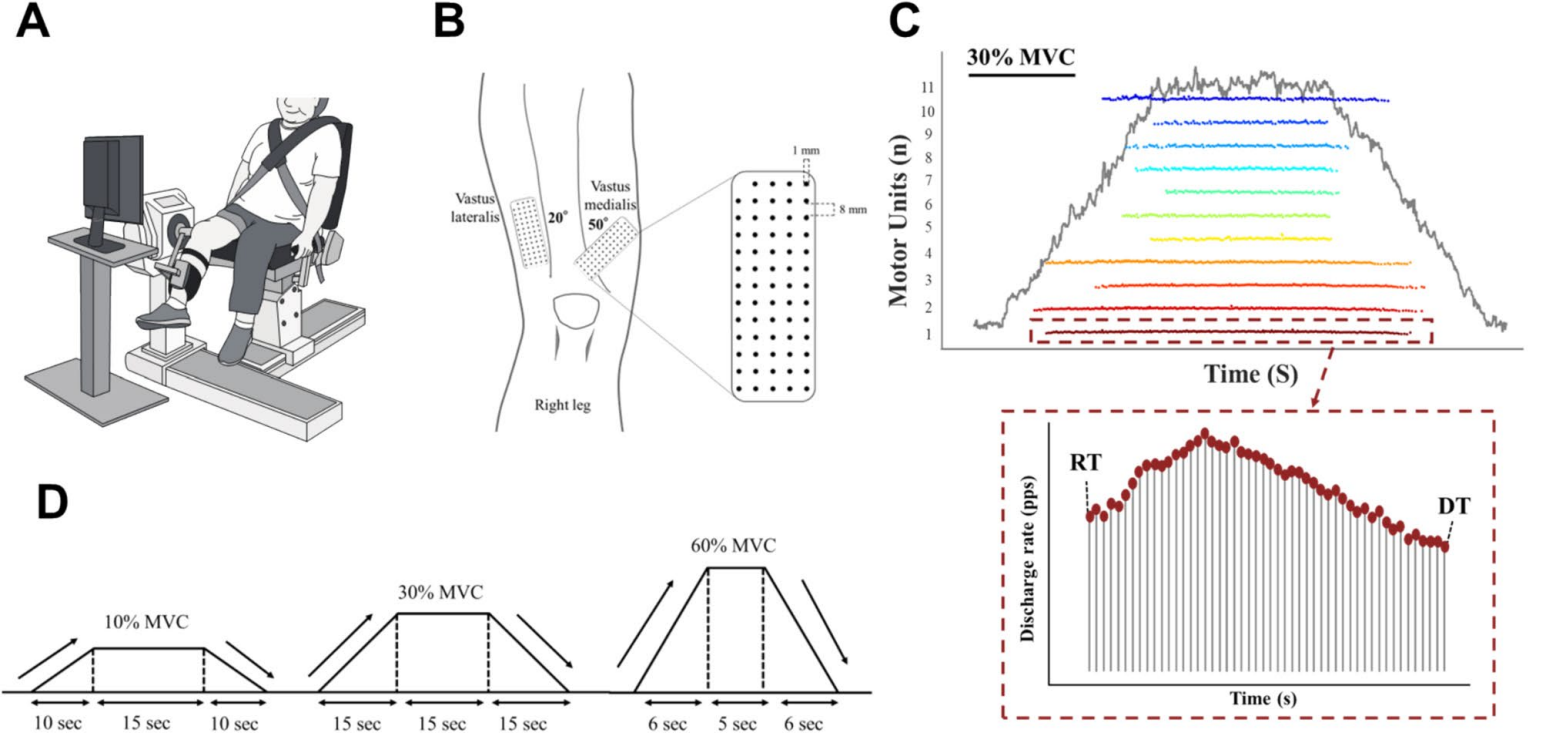
Experimental setup. **A** Each participant was seated on an isokinetic dynamometer with the knee positioned at 45° while performing the motor task. **B** Two grids, each containing 64 electrodes, were placed over the vastus lateralis (VL) and vastus medialis (VM) at angles of 20° and 50°, respectively, relative to the reference lines. **C** Representative images showing high-density surface electromyography decomposition in early-stage knee osteoarthritis (KOA). Recruitment (RT) and derecruitment (DT) are calculated as the force values at which motor units are activated and deactivated, corresponding to the first and last spike. **D** Participants completed three submaximal voluntary contractions

The procedures used for force and HD-SEMG signal recordings were consistent with those detailed in previous studies [25]. The participants were seated comfortably and connected to an isokinetic dynamometer (BIODEX System 4; BIODEX Company, USA), with the knee joint maintained at 45° of flexion (Fig. 1A). HD-SEMG signals were captured from the VM and VL muscles via two bidimensional grids, each comprising 64 electrodes (5 columns × 13 rows; gold-coated; 1 mm diameter; 8 mm interelectrode distance; OT Bioelettronica, Turin, Italy). The placement and orientation of the electrode grids were optimized according to established guidelines and fine-tuned following the methodology described in our prior research [25] (Fig. 1B). After the skin was prepared by shaving and cleansing with 80% ethanol, the electrode grids were attached to disposable biadhesive foam layers (FOA08MM1305, OT Bioelettronica, Italy) [20], and firm skin‒electrode contact was ensured by filling the foam layer holes with conductive paste (Elefix ZV-181E, NIHON KOHDEN, Tokyo, Japan). The ground electrode was placed on the ipsilateral ankle. The monopolar HD-SEMG signals were digitized with a 16-bit analogue-to-digital converter (Quattrocento, OT Bioelettronica, sampling at 2048 Hz, or Mouvi + Pro, OT Bioelettronica, sampling at 2000 Hz), amplified with a gain of 150, and band-pass filtered offline between 10 and 500 Hz. Analysis of the force and EMG signals was conducted using MATLAB software (MATLAB 2023b, MathWorks GK, MA, USA).

### Experimental Protocol for Assessing Neuromuscular Characteristics

Following a standardized warm-up, the participants completed two to three trials, each lasting approximately 3–5 s, during which they were verbally encouraged to achieve their maximal voluntary isometric force (MVC) via unilateral knee extension. The contraction level for submaximal ramp-up contractions was determined on the basis of the peak knee extension torque measured at baseline. After a rest of approximately 5 minutes, the participants were instructed to perform submaximal trapezoidal contractions at three distinct force levels (2 × 10%, 30%, and 60% MVC). These contractions involved a ramp-up phase (linear force increase at 5% MVC per second), a plateau phase (constant force at the target for 15 seconds at 10% and 30% MVC and for 5 seconds at 60% MVC), and a ramp-down phase (linear force decrease at 5% MVC per second) [19, 26] (Fig. 1C, D). During these tasks, the participants exerted force with their knee extensors, aiming to match a visual template of the trapezoidal force pattern displayed on a monitor positioned 1 meter away. A rest period of three minutes was provided between each submaximal contraction. HD-SEMG signals were recorded from the VM and VL muscles on the side affected by KOA. The participants were also instructed to avoid strenuous exercise and caffeine consumption for 48 hours prior to the test.

### Data Processing

Monopolar EMG signals were initially band-pass filtered and visually inspected for noise and artifacts. Channels with noise or artifacts were excluded from further analysis. A validated convolutive blind source separation technique was employed to decompose the HD-SEMG recordings into the discharge timings of individual MUs Following decomposition [27, 28], all identified MU spike trains underwent manual verification by a single investigator. Individual MUs were identified using DEMUSE software (v. 6.0; The University of Maribor, Slovenia). MU spike trains with interspike intervals of less than 33.3 ms or greater than 250 ms (corresponding to DR (pulse per second, pps) exceeding 30 pps or below 4 pps, respectively) and those with a low pulse-to-noise ratio (< 30 dB, indicating an DR identification accuracy of less than 90%) were excluded from the analysis [26]. The instantaneously identified DR was determined by calculating the time interval between consecutive spikes, and the mean DR was subsequently computed during for each phase of trapezoidal contraction. The RT and derecruitment threshold (DT) for each MU were defined as the relative force levels (%MVC) at which the first and last action potentials were discharged, respectively. Additionally, the coefficient of variation (CV) of force (calculated as the standard deviation divided by the mean, multiplied by 100) was determined for each level of sustained submaximal contraction.

### Statistical Analyses

Data normality was assessed via the Shapiro‒Wilk test. Given that the data followed a normal distribution, parametric analyses were conducted to compare variables such as age, height, weight, K/L grade, knee extension torque, subcutaneous fat, and numerical rating scale scores between the early and severe KOA groups via unpaired *t* tests. For variables that did not meet the normality assumption, a nonparametric approach was employed, specifically, a generalized linear mixed-effects model with both random intercepts and slopes (lme4 package of R). This model was used to analyze the CV of force, the mean DR, and the RT. The explanatory variables included a comparison of the CV of force, DR, RT, and DT across different groups (early KOA vs. severe KOA), muscles (VM vs. VL), and contraction levels (10%, 30%, and 60% MVC). The Bonferroni correction was applied to adjust for multiple comparisons, ensuring that the effects of multiple testing were adequately controlled. Repeated-measures correlation (using the rmcorr package of R) was performed to determine the DR and RT. Correlations between knee extension torque and the CSA, CTV, RT and DR in each group and muscle type were calculated via Pearson’s correlation coefficient. All statistical analyses were performed using R version 4.2.3 (The R Foundation for Statistical Computing, Austria). Statistical significance was set at *p* < 0.05, and estimates were presented with 95% confidence intervals (CI).

## Results

### Participant Characteristics

Thirty female participants were recruited into two groups (Early and Severe KOA) which were well balanced with regard to age, weight, and height (Table 1). Significant between-group differences in the K/L grade and KSS were found (*p* < 0.001). The CSA of the VM muscle was significantly smaller in the severe KOA group than in the early KOA group (severe KOA: 10.9 ± 3.9 cm^2^; early KOA: 7.9 ± 2.4 cm^2^; *p* = 0.021) (Table 2). There was no significant difference in the CTV of the VM or VL muscle between the groups (*p* > 0.05). The CV of force did not show a significant group × contraction level × MVC interaction (*p* = 0.964), main effect of group (*p* = 0.893), contraction level (*p* = 0.883), and MVC (*p* = 0.164) (Supplementary Result 1).

**Table 1:** Characteristics of participants.

Characteristics	Early KOA	Severe KOA	*p*-value
N (Females)	15	15	NA
Age (years)	74.3 ± 5.5	75.2 ± 6.3	0.670
Height (cm)	153.8 ± 6.4	151.0 ± 8.7	0.327
Weight (kg)	58.2 ± 10.9	57.5 ± 10.0	0.863
Kellgren Lawrence grade	I: 8, II: 7	III: 6, IV: 9	**0.001**
Knee Society Score	73.7 ± 8.1	26.1 ± 8.9	**0.001**
Knee extension torque (Nm)	64.3 ± 25.1	68.3 ± 33.7	0.711
Subcutaneous tissue (mm)	19.2 ± 5.4	19.7 ± 5.3	0.820
Numerical Rating Scale	1.4 ± 0.9	1.7 ± 0.9	0.387

Variables are presented as the mean ± SD. *KOA* knee osteoarthritis

**Table 2:** Comparison of the CSA and CTV of the VM and VL between the early and severe KOA groups.

	Early KOA	Severe KOA	*p*-value
VM CSA (cm^2^)	10.9 ± 3.9	7.9 ± 2.4	**0.021**
VL CSA (cm^2^)	15.1 ± 3.4	14.0 ± 3.3	0.391
VM CTV (HU)	45.6 ± 9.4	41.6 ± 13.7	0.312
VL CTV (HU)	43.9 ± 12.3	45.4 ± 12.4	0.737

Variables are presented as the mean ± SD. *KOA* knee osteoarthritis, *VM* vastus medialis, *VL* vastus lateralis, *CSA* cross-sectional area, *CTV* computed tomography attenuation value

### MU Decomposition

We identified a total of 682 MUs (early KOA: 348 MUs; severe KOA: 334 MUs) that were considered for further analysis. The distribution and number of MUs for each group, muscle and contraction level are shown according to the RT (Supplementary Result 1).

### Properties of MUs

**Fig. 2:**
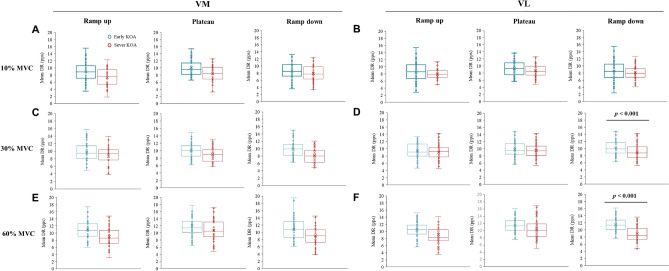
Differences in the DR. Comparison between the vastus medialis (VM) (left side) and vastus lateralis (VL) (right side) muscles in the early and severe knee osteoarthritis (KOA) groups. The motor unit (MU) discharge rate (DR) is displayed separately for each phase of the trapezoidal contraction (i.e., ramp-up, plateau, ramp-down) and for each contraction level. This included 10% maximal voluntary contraction (MVC) in panels **A** and **B**, 30% MVC in panels **C** and **D**, and 60% MVC in panels **E** and **F**

The participant-specific values of the mean DR obtained from the whole trapezoidal contraction are displayed for each contraction level, muscle and group in Fig. 2. No significant group × contraction level interactions were found for each phase of the trapezoidal contraction or for the VM muscle (ramp-up: *p* = 0.492; plateau: *p* = 0.302; ramp-down: *p* = 0.145). A significant main effect of group was found for the VL muscle, which was significantly lower in the severe KOA group than in the early KOA group during the ramp-up phase (*p* = 0.004). Significant group × contraction level interactions were found for the ramp-down phase of the VL muscle at 30 and 60% MVC (*p* < 0.001). Specifically, during the ramp-down phase, the DR of the VL muscle in the severe KOA group was significantly lower than that in the early KOA group at 30% MVC (early KOA: 95% CI 9.687 to 10.731 pps; severe KOA: 95% CI 8.477 to 9.402 pps; *p* = 0.001; Fig. 2D) and 60% MVC (early KOA: 95% CI 10.159 to 12.725 pps; severe KOA: 95% CI 7.623 to 10.385 pps; *p* = 0.001; Fig. 2F). Compared with those in the early KOA group, the RT and DT of the VM and VL muscles in the severe KOA group were similar (*p* > 0.05, Supplementary Result 2).

### Repeated-Measures Correlation Coefficients

**Fig. 3:**
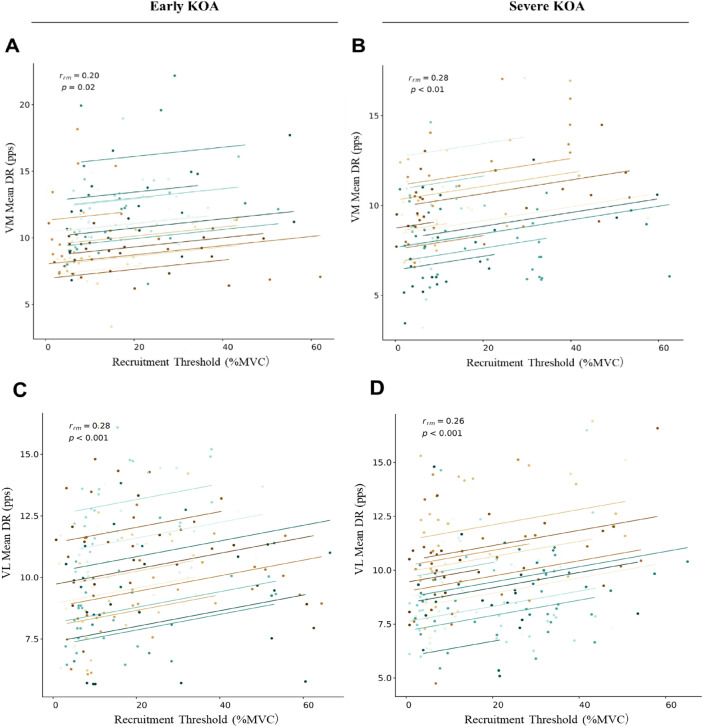
Relationship between the RT and the DR. Repeated-measures correlation coefficients of the motor unit recruitment threshold (RT) and discharge rate (DR) in the vastus medialis and vastus lateralis muscles for participants from both the early and severe knee osteoarthritis (KOA) groups

The RT was significantly related to the DR of the VM muscle obtained during the plateau phase of trapezoidal contraction in both groups (early KOA; *r*_*rm*_ = 0.20; *p* = 0.02; severe KOA; *r*_*rm*_ = 0.28; *p* < 0.010; Fig. 3A, B). The RT was significantly related to the DR of the VL muscle obtained during the plateau phase of contraction in both groups (early KOA; *r*_*rm*_ = 0.28; *p* < 0.001; severe KOA; *r*_*rm*_ = 0.26; *p* < 0.001; Figure 3C, D).

### Relationships Between the CSA, CTV, RT, and DR and Knee Extension Torque

**Fig. 4:**
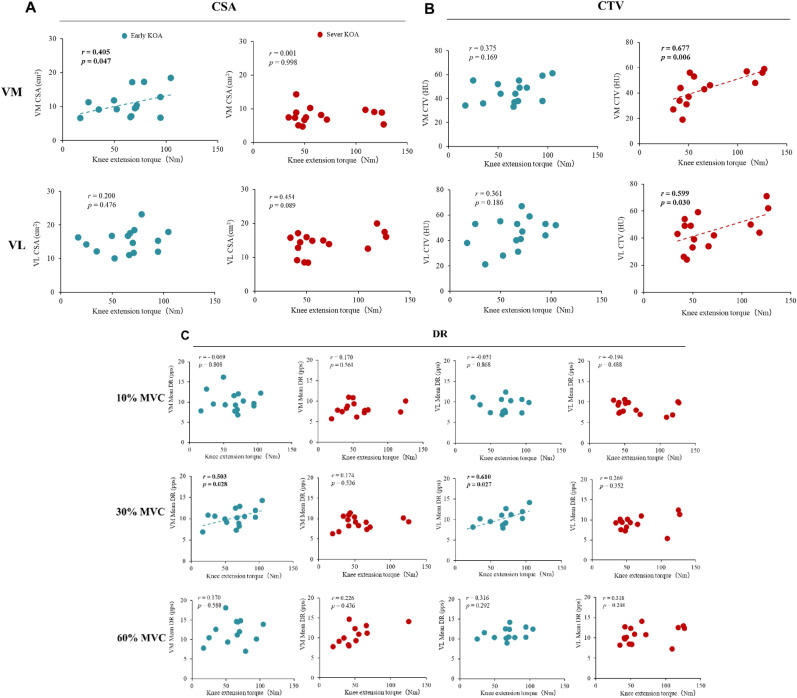
Relationships among knee extension torque, CSA, CTV and DR. Significant correlations were observed between knee extension torque and the cross-sectional area (CSA) of the vastus medialis (VM) in the early knee osteoarthritis (KOA) group (**A**) and between knee extension torque and the computed tomography attenuation value (CTV) of the VM and vastus lateralis (VL) in the severe KOA group (**B**). MUs were clustered according to the contraction level. Significant correlations were observed between the knee extension torque and discharge rate (DR) in the VM and VL muscles in the early KOA group at 30% maximal voluntary contraction (MVC) (**C**)

In the early KOA group, knee extension torque was significantly correlated with the CSA of the VM muscle (*r* = 0.405, *p* = 0.047; Fig. 4A) and the DR obtained at 30% MVC of the VM muscle (*r* = 0.503, *p* = 0.028; Fig. 4C). In the severe KOA group, knee extension torque was significantly correlated with the CTV of the VM muscle (*r* = 0.677, *p* = 0.006; Fig. 4B) and the CTV of the VL muscle (*r* = 0.599, *p* = 0.030; Fig. 4B), and no significant correlations between the CSA, DR, and knee extension torque were found (*p* > 0.05). Specifically, no significant correlations between the MVC and RT for the VM or VL muscles were found (*p* > 0.05, Supplementary Result 3).

## Discussion

This study compared the firing properties of individual MUs in the VM and VL muscles during submaximal isometric contractions between the early and severe KOA groups via HD-SEMG. We found that (1) the firing pattern hierarchy was absent in the early and severe KOA groups (Fig. 3) and that (2) the severe KOA group showed a significantly smaller CSA of the VM than the early KOA group, but the neural system changes in the DR were not affected. (3) Severe KOA group showed a significantly lower frequency of the ramp-up DR of the VL muscle than the early KOA group at each contraction level. (4) The maximal muscle strength of the early KOA group was significantly correlated with the CSA of the VM muscle and the DR obtained at 30% MVC of the VM muscle. In addition, the maximal muscle strength of the severe KOA group was significantly correlated with the CTV of the VM and VL muscles. These results partly support our hypothesis that early recruited MUs do not fire faster than later-recruited ones, and that MU DR and muscle quality are associated with maximal strength in patients with severe KOA. Furthermore, the use of HD-SEMG in this study enabled non-invasive, high-resolution assessment of individual DR behaviours, contributing novel insights into the neuromuscular adaptations in KOA. This emerging technique offers the potential to detect early motor control impairments that may precede overt muscle atrophy, thereby providing a foundation for earlier and more targeted interventions.

The early and severe KOA groups were found to have a significant positive correlation between the DR and RT. According to the size principle, MUs with smaller motoneuron cell sizes are recruited first, followed by those with larger motoneuron cell sizes. Typically, MUs with a low RT fire at higher frequencies, whereas those with a high RT fire at lower frequencies during submaximal contraction. This hierarchical firing pattern is commonly referred to as the ‘onion skin phenomenon’ [29]. However, the DR patterns of older adults and those with presarcopenia differ from those of young, healthy adults, often resulting in a less distinct hierarchical pattern [21, 30]. Previous studies have shown that during ramp contractions at low force levels, the DR of elderly individuals are significantly lower than those of the younger individuals [30, 31]. This reduction in DR among elderly individuals has been attributed to the presence of a greater proportion of type I muscle fibers in aging muscles, which is the result of an increased innervation ratio and the selective atrophy or loss of type II fibers with age [32, 33]. Consequently, the decline in DR in older adults is considered a neurophysiological adaptation aimed at efficiently activating type I muscle fibers while minimizing high-frequency fatigue. The DR of the VM and VL muscles during 30% MVC tasks averaged 10–15 pps in young and elderly individuals [34, 35], whereas the results of this study showed that the DR of the VM and VL muscles during 10% and 30% MVC tasks was less than 10 pps. These findings suggest that the DR and RT are positively correlated in the KOA groups, as the DR is reduced with a low RT of the VM and VL muscles. Therefore, neuromuscular degeneration may occur not only in patients with severe KOA but also in patients with early KOA.

The results of this study revealed that the VM muscle of the patients in the severe KOA group had a significantly smaller CSA than did the early KOA group, regardless of neuromuscular characteristics. A previous study reported that patients with KOA have significantly thinner muscle thickness in the VM muscle and no significant difference in muscle thickness in the VL muscle compared with those of healthy elderly patients [4], and the results obtained for these muscles are consistent with the results of this study. In addition, no significant difference in the DR of the VM muscle has been reported between participants with presarcopenia and young adults, although participants with sarcopenia have significantly lower skeletal muscle mass [36]. In addition, the muscle fibers of the VM include vastus medialis longus and vastus medialis obliquus fibers [37]. Therefore, future studies may be able to evaluate the neuromuscular activity of the VM muscle in more detail by accounting for the longitudinal and oblique fibers of the VM muscle and measuring them.

The results of this study revealed that the severe KOA group had a significantly lower DR of the VL muscle at ramp-up, regardless of the force level, than did the early KOA group. The skeletal muscle architecture acts to receive inputs from the nervous system, and each muscle plays a role in optimizing its function in the body [38]. Although both are pinnate muscles, the physiological CSA of the VL muscle is approximately two times greater than that of the VM muscle, which means that the VL muscle has a beneficial fiber arrangement to produce force [39, 40]. Thus, patellar tracking may be stabilized by maintaining intermuscular balance between the VM and VL muscles during knee joint extension movements. Since patients with severe OA exhibited muscle atrophy of the VM in this study, the reduced DR of the VL muscle may be a neuromuscular control mechanism to maintain intermuscular balance during ramp-up regardless of the task condition. In addition, during the ramp-down phase of the MU, the DR of the VL muscle was significantly lower in the severe KOA group than in the early KOA group at 30% and 60% MVC. The maximal voluntary muscle strength of healthy young adults was maintained during the 70% MVC task, but the DR of the VL muscle was reported to be lower during eccentric contraction than during concentric contraction [35]. In addition, an increase in the DR of the VM and rectus femoris muscles during knee extension tasks was reported in a study in which the VL muscle was fatigued by electrical muscle stimulation [41]. These findings suggest that the VM and rectus femoris muscles, which are both muscles of the quadriceps, may compensate for the reduced DR of the VL muscle to maintain maximal voluntary muscle strength during the knee extension task in the context of overload.

The maximal muscle strength of the early KOA group was significantly correlated with the CSA of the VM muscle and the DR obtained at 30% MVC of the VM muscle. In addition, the maximal muscle strength of the severe KOA group was significantly correlated with the CTV of the VM and VL muscles. A previous CT study reported a correlation between the CSA of the VM muscle and knee extension muscle strength in an analysis of the CSA and CTV of each quadriceps muscle in older adults [42]. Skeletal muscle mass and strength decrease with age [42, 43], and the muscle quality also decreases with age [42, 44]. Previous studies have reported that muscle quality is lower in older patients with KOA [4], but no significant differences were observed based on KOA severity [45, 46], which is consistent with the findings of the present study. On the other hand, it has been reported that increased non-contractile skeletal muscle tissue, such as intramuscular fat, results in overestimation of the actual muscle contractile tissue [24]. Therefore, it is possible that the maximum knee joint extension muscle strength in patients with severe KOA does not correlate with the muscle quantity of the VM or VL muscle. In addition, patients with KOA exhibit muscle atrophy according to the muscle thickness of the VM muscle but no significant difference in the VL muscle [46], and the results of that previous study are consistent with the results of this study. Patients with KOA are known to exhibit increased knee adduction during gait, potentially leading to greater mechanical stress on the VL muscle. To compensate for the reduced activation of the VM muscle, patients with knee OA tend to increase the activation of the rectus femoris and VL muscles during gait [47, 48]. Therefore, the increased muscle activity observed during gait may help maintain both the quantity and quality of the VL muscle in patients with severe KOA.

These findings indicate the potential benefit of tailoring rehabilitation programs according to disease severity. For patients with early KOA, interventions focusing on improving neuromuscular control such as motor unit recruitment training and neuromuscular electrical stimulation [49, 50] may help preserve muscle function and slow disease progression. In contrast, patients with severe KOA may benefit more from resistance training programs [51] aimed at enhancing residual motor unit activation and compensating for lost muscle fibers. In addition, by focusing on both early and severe KOA stages in female patients a population at high risk for disease progression this study emphasizes the importance of early neuromuscular assessment and intervention strategies. Our findings support the potential for developing personalized rehabilitation approaches that account for disease stage and sex-specific neuromuscular characteristics, ultimately improving clinical outcomes in KOA.

This study has several limitations. First, this study recruited only females with KOA. MU DR behavior changes with age [22], and there are sex differences in these changes [26]. Therefore, the results may not be generalizable to healthy adults and males with KOA. Future studies should include male participants and healthy older adults to better understand sex-related and disease-specific changes in MU behaviors. Second, the recruited population consisted of individuals with minimal knee pain and preserved independence in daily activities. Since DR can be influenced by pain severity [52], the present findings may not be fully applicable to individuals with KOA experiencing moderate to severe pain or greater functional limitations. Future studies should include participants with a broader range of pain severity and functional status to clarify how pain-related factors modulate DR patterns. Third, our analyses were restricted to two quadriceps muscles, the VM and VL. While these muscles are critical for knee joint stabilization and are commonly studied in KOA, it is possible that other components of the quadriceps, such as the rectus femoris or vastus intermedius, also contribute to motor control changes. Future studies should also evaluate the potential contribution of other quadriceps muscles to knee extensor torque generation. Fourth, the sample size was modest, which may limit the statistical power to detect subtle differences in MU behaviours, may have resulted in a type II error. These non-significant results may need to be clinically interpreted with caution. Finally, this study did not consider psychological, hormonal, or pain-related factors (e.g., motivation, anxiety, pain threshold, analgesic use), which may affect voluntary activation and EMG accuracy. These variables may act as potential confounders and introduce inter-individual variability in the results. Future studies should incorporate validated assessments of psychological state and other biopsychosocial variables to more comprehensively characterize MU behaviours in KOA.

## Conclusions

In conclusion, patients with severe KOA, characterized by a smaller CSA of the VM muscle, displayed distinct DR patterns at various contraction levels compared to the early KOA group. These findings suggest the existence of a compensatory strategy that may help offset the reduction in muscle mass and maintain knee extensor strength. The VM and VL muscles fired without a hierarchical pattern in the early and severe KOA groups. These findings suggest that neuromuscular degeneration is not limited to advanced KOA stages but may already begin in early KOA, highlighting the importance of early neuromuscular assessment and intervention.
